# Tumor Necrosis Factor-α-Induced C-C Motif Chemokine Ligand 20 Expression through TNF Receptor 1-Dependent Activation of EGFR/p38 MAPK and JNK1/2/FoxO1 or the NF-κB Pathway in Human Cardiac Fibroblasts

**DOI:** 10.3390/ijms23169086

**Published:** 2022-08-13

**Authors:** Chuen-Mao Yang, Chien-Chung Yang, Wun-Hsin Hsu, Li-Der Hsiao, Hui-Ching Tseng, Ya-Fang Shih

**Affiliations:** 1 Department of Pharmacology, College of Medicine, China Medical University, Taichung 40402, Taiwan; 2 Ph.D. Program for Biotech Pharmaceutical Industry, China Medical University, Taichung 40402, Taiwan; 3 Department of Post-Baccalaureate Veterinary Medicine, College of Medical and Health Science, Asia University, Wufeng, Taichung 41354, Taiwan; 4 Department of Traditional Chinese Medicine, Chang Gung Memorial Hospital at Tao-Yuan, Kwei-San, Tao-Yuan 33302, Taiwan; 5 School of Traditional Chinese Medicine, College of Medicine, Chang Gung University, Kwei-San, Tao-Yuan 33302, Taiwan

**Keywords:** TNF-α, CCL20, cardiac fibroblasts, cardiac inflammation

## Abstract

Tumor necrosis factor (TNF)-α is involved in the pathogenesis of cardiac injury, inflammation, and apoptosis. It is a crucial pro-inflammatory cytokine in many heart disorders, including chronic heart failure and ischemic heart disease, contributing to cardiac remodeling and dysfunction. The implication of TNF-α in inflammatory responses in the heart has been indicated to be mediated through the induction of C-C Motif Chemokine Ligand 20 (CCL20). However, the detailed mechanisms of TNF-α-induced CCL20 upregulation in human cardiac fibroblasts (HCFs) are not completely defined. We demonstrated that in HCFs, TNF-α induced CCL20 mRNA expression and promoter activity leading to an increase in the secretion of CCL20. TNF-α-mediated responses were attenuated by pretreatment with TNFR1 antibody, the inhibitor of epidermal growth factor receptor (EGFR) (AG1478), p38 mitogen-activated protein kinase (MAPK) (p38 inhibitor VIII, p38i VIII), c-Jun amino N-terminal kinase (JNK)1/2 (SP600125), nuclear factor kappaB (NF-κB) (helenalin), or forkhead box O (FoxO)1 (AS1841856) and transfection with siRNA of TNFR1, EGFR, p38α, JNK2, p65, or FoxO1. Moreover, TNF-α markedly induced EGFR, p38 MAPK, JNK1/2, FoxO1, and NF-κB p65 phosphorylation which was inhibited by their respective inhibitors in these cells. In addition, TNF-α-enhanced binding of FoxO1 or p65 to the CCL20 promoter was inhibited by p38i VIII, SP600125, and AS1841856, or helenalin, respectively. Accordingly, in HCFs, our findings are the first to clarify that TNF-α-induced CCL20 secretion is mediated through a TNFR1-dependent EGFR/p38 MAPK and JNK1/2/FoxO1 or NF-κB cascade. We demonstrated that TNFR1-derived EGFR transactivation is involved in the TNF-α-induced responses in these cells. Understanding the regulation of CCL20 expression by TNF-α on HCFs may provide a potential therapeutic strategy in cardiac inflammatory disorders.

## 1. Introduction

Tumor necrosis factor (TNF)-α levels are increased in patients with dilated cardiomyopathy and ischemic heart disease but not expressed in the normal heart. The severity of heart failure (HF) and survival rate correspond to the levels of TNF-α in the serum of patients [1,2]. TNF-α is a pro-inflammatory cytokine that activates innate immune pathways and triggers inflammatory responses through two transmembrane receptor subtypes: TNFR1 and TNFR2. TNFR-induced remodeling responses are accompanied by cardiac inflammation through NF-κB activation, inflammatory cytokine expression, mitogen-activated protein kinases (MAPKs) activity, and apoptosis [3,4]. An early study also demonstrated that the expressions of TNF-α and soluble TNFRs are consistently produced in dilated cardiomyopathy and ischemic heart disease [5]. TNF-α has an important role in myocardial remodeling [6]. In vivo study using the model of diabetic cardiomyopathy demonstrated that the inhibition of TNF-α can relieve left ventricle dysfunction by reducing myocardial inflammation and cardiac fibrosis mediated by TNF-α responses [7].

Chemokines are a large family of key mediators during immunoregulatory and pro-inflammatory responses. Chemokines are classified as cysteine–cysteine (C–C) and cysteine–noncysteine–cysteine (C–X–C). Chemokine (C-C motif) ligand 20 (CCL20) is also known as macrophage inflammatory protein-3α or liver activation regulated chemokine, which is a member of the chemokine family. CCL20 elicits its effects by activating the chemokine receptor CC chemokine receptor 6 (CCR6), which is the only chemokine known to interact with CCR6 [8]. It has been uncovered that the serum level of CCL20 is correlated with ischemic heart disease [9]. Recently, in clinical reports, it was found that CCL20 could be a biomarker to predict the severity and prognosis of HF [10]. Moreover, TNF-α is critically involved in many pathogeneses of inflammatory diseases through the induction of CCL20 expression [11,12]. Therefore, we hypothesize that upregulation of CCL20 induced by TNF-α may be crucial in cardiac inflammation. The CCL20 promoter region contains a nuclear factor kappaB (NF-κB) binding site [13]. Moreover, forkhead box O (FoxO)1 could potentiate transcription of CCL20 by promoting the binding activity of p65/p50 heterodimer to their functional NF-κB site in the CCL20 promoter in TNF-α-treated HepG2 cells [14]. Previous studies indicate that receptor tyrosine kinases (RTKs) or mitogen-activated protein kinases (MAPKs) are involved in TNF-α-induced responses in various types of cells [4,15,16,17]. Thus, the present study aims to dissect the roles of these signaling components and transcription factors in regulating the expression of CCL20 in human cardiac fibroblasts (HCFs).

The majority of cells in the heart consist of fibroblasts and myocytes. Cardiac fibroblasts play a prominent role in maintaining normal cardiac structure and function and participate in cardiac development and heart remodeling during pathological conditions [18]. These cells participate in cell–cell communication with myocytes and other fibroblasts, as well as with endothelial cells and the synthesis and deposition of the extracellular matrix (ECM), which affect the electrophysiological properties and secretion of growth factors and cytokines as well as angiogenesis [19]. Therefore, fibroblasts contribute to the deposition of the excessive fibrotic ECM and directly cause proliferation and hypertrophy of cardiomyocytes by paracrine mechanisms [18,20,21]. Cardiac fibroblasts secrete pro-inflammatory cytokines with chemotactic effects and may act as sentinel cells activated by inflammatory mediators such as TNF-α or mechanical stress [22,23]. Therefore, understanding the role and possible mechanisms of HCFs in cardiac inflammation is an important issue.

However, the molecular mechanisms of TNF-α-induced CCL20 expression in HCFs remain unclear. The objective of this study is to address the mechanisms underlying TNF-α-induced CCL20 expression in HCFs. This study clarifies the role of CCL20 in cardiac inflammation and the mechanisms of TNF-α-induced CCL20 expression. Our results suggest that TNF-α-induced CCL20 expression is, at least partially, mediated through binding with TNFR1 to activate epidermal growth factor receptor (EGFR)/p38 MAPK and c-Jun amino N-terminal kinases (JNK)1/2/forkhead box O (FoxO)1 pathways or stimulate NF-κB signaling in HCFs. These findings can provide potential therapeutic strategies for treating inflammatory heart diseases.

## 2. Results

### 2.1. TNF-α Induces CCL20 Protein Secretion and mRNA Expression in HCFs

**Figure 1:**
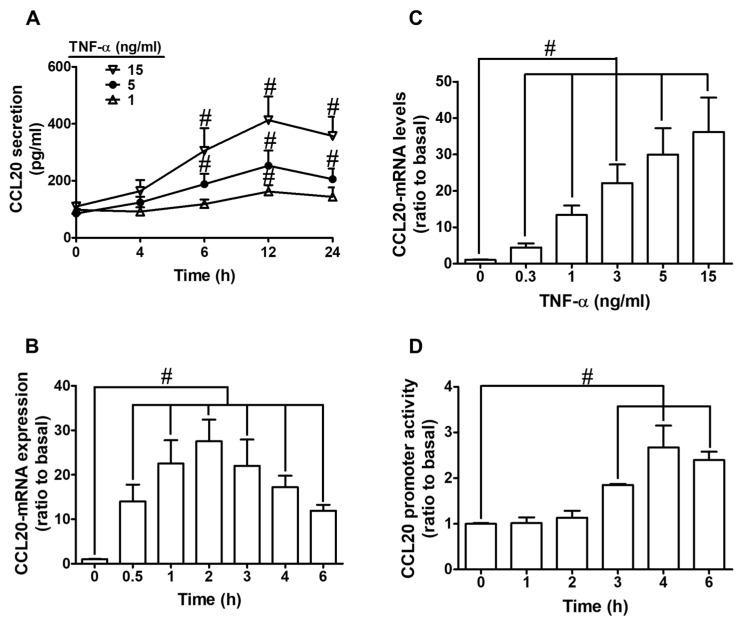
TNF-α induces CCL20 protein secretion and mRNA expression in HCFs. (**A**) Cells were incubated with TNF-α for the indicated time intervals (4, 6, 12, and 24 h). The conditioned media were utilized to determine the CCL20 level via an ELISA kit. (**B**,**C**) The mRNA levels of CCL20 were determined by real-time PCR. (**B**) Cells were incubated with TNF-α (5 ng/mL) for the indicated time intervals (0.5, 1, 2, 3, 4, and 6 h. (**C**) Cells were incubated with 0.3, 1, 5, and 15 ng/mL TNF-α for 2 h. (**D**) Cells were incubated with TNF-α (5 ng/mL) for the indicated time intervals (1, 2, 3, 4, and 6 h). The promoter activity of CCL20 was determined by a promoter assay kit. Data are expressed as mean ± S.E.M. of three independent experiments (*n* = 3). ^#^ *p* < 0.05, as compared with the cells exposed to vehicle alone.

To evaluate the effect of TNF-α on CCL20 production, HCFs were treated with various concentrations of TNF-α (0, 1, 5, and 15 ng/mL) for the indicated time intervals (0, 4, 6, 12, and 24 h). As shown in Figure 1A, we found that TNF-α time- and concentration-dependently increased CCL20 protein secretion. The levels of CCL20 had a significant increase within 6 h; a maximal response was observed within 12 h and slightly declined within 24 h. To determine whether TNF-α promoted CCL20 expression through transcriptional regulation, HCFs were treated with various concentrations of TNF-α (0, 0.3, 1, 3, 5, 15 ng/mL) for 2 h or 5 ng/mL TNF-α for the indicated time intervals (0, 0.5, 1, 2, 3, 4, 6 h). As shown in Figure 1B, TNF-α (5 ng/mL) caused CCL20 mRNA expression in a time-dependent manner, with a maximal response within 2 h. In addition, TNF-α also induced CCL20 mRNA expression in a concentration-dependent manner, with a maximal response at 15 ng/mL of TNF-α (Figure 1C). These results suggest that TNF-α time- and concentration-dependently promoted CCL20 secretion through the induction of CCL20 mRNA and protein expression. Further, the transcriptional regulation of CCL20 gene expression stimulated by TNF-α was disclosed by a promoter activity assay. As shown in Figure 1D, TNF-α (5 ng/mL) enhanced CCL20 promoter activity and reached a maximal response within 4 h. Together, these results suggest that TNF-α-induced CCL20 secretion is mediated by increasing gene transcription in HCFs.

### 2.2. TNF-α-Induced CCL20 Expression via TNFR1 in HCFs

**Figure 2:**
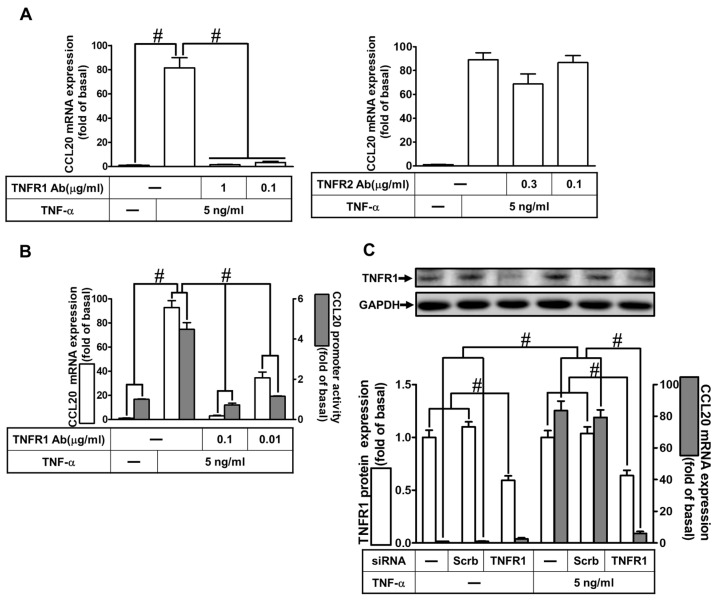
TNF-α-induced CCL20 expression via TNFR1 in HCFs. (**A**) Cells were pretreated with 1 and 0.1 μg/mL TNF receptor 1 antibody (TNFR1 Ab) or TNFR2 Ab for 1 h, and then incubated with 5 ng/mL TNF-α for 2 h. The mRNA levels of CCL20 were determined by real-time PCR. (**B**) Cells were pretreated with 0.1 and 0.01 μg/mL TNFR1 Ab for 1 h, and then incubated with 5 ng/mL TNF-α for the indicated time. The mRNA levels and promoter activity of CCL20 were determined by real-time PCR (2 h) and promoter assay (4 h), respectively. (**C**) Cells were transfected with scrambled or TNFR1 siRNA and then incubated with TNF-α for 2 h. The mRNA levels of CCL20 were determined by real-time PCR. The protein levels of TNFR1 were determined by Western blot with GAPDH as a loading control. Data are expressed as mean ± S.E.M. of three independent experiments (*n* = 3). ^#^ *p* < 0.05, as compared with the cells exposed to vehicle alone; or significantly different as indicated.

TNF-α has been shown to regulate cellular functions through TNFR1 or TNFR2 in different types of cells [3,4]. To determine which type of TNFRs was involved in TNFR-α induced CCL20 expression, HCFs were pretreated with TNF receptor 1 neutralized antibody (TNFR1 nAb) or TNFR2 nAb for 1 h and then incubated with TNF-α for 12 h. As shown in Figure 2A, TNF-α-induced CCL20 mRNA expression was significantly attenuated by pretreatment with TNFR1 nAb in a concentration-dependent manner but not by TNFR2 nAb. Further, pretreatment with TNFR1 nAb also inhibited the TNF-α-induced mRNA expression and promoter activity of CCL20 in HCFs (Figure 2B). To ascertain the role of TNFR1 in CCL20 expression, as shown in Figure 2C, transfection with TNFR1 siRNA downregulated the TNFR1 protein expression and also attenuated the TNF-α-induced CCL20 mRNA expression. These results suggest that TNF-α-induced CCL20 expression is mediated through the TNFR1 in HCFs.

### 2.3. Involvement of EGFR in TNF-α-Induced CCL20 Expression

**Figure 3:**
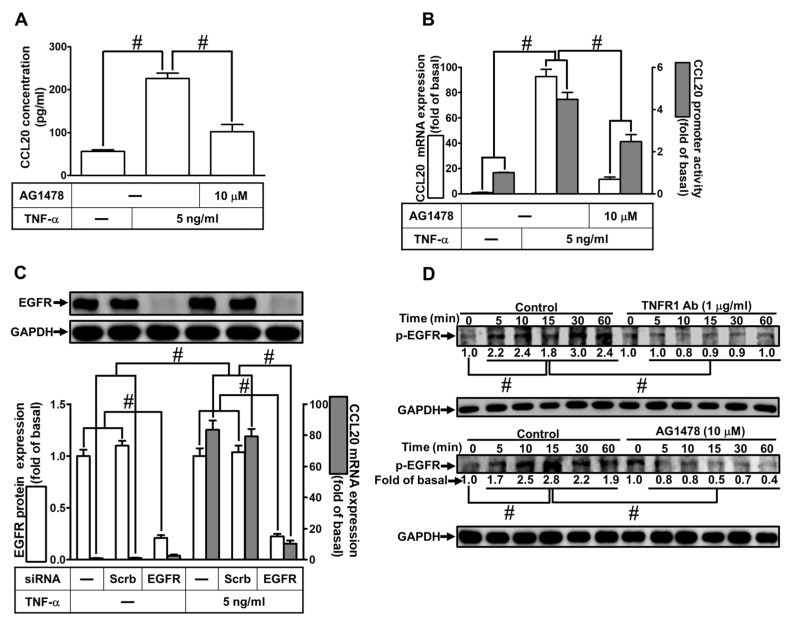
Involvement of EGFR in TNF-α-induced CCL20 expression. (**A**) Cells were pretreated with 10 μM AG1478 for 1 h and then incubated with 5 ng/mL TNF-α for 12 h. The conditioned media were utilized to determine the CCL20 level via an ELISA kit. (**B**) Cells were pretreated with 10 μM AG1478 for 1 h and then incubated with 5 ng/mL TNF-α for the indicated time. The mRNA levels and promoter activity of CCL20 were determined by real-time PCR (2 h) and promoter assay (4 h), respectively. (**C**) Cells were transfected with scrambled or EGFR siRNA and then incubated with TNF-α for 2 h. The mRNA levels of CCL20 were determined by real-time PCR. The protein levels of EGFR were determined by Western blot with GAPDH as a loading control. (**D**) Cells were pretreated without or with 1 μg/mL TNFR1 Ab or 10 μM AG1478 for 1 h and then treated with TNF-α for the indicated time (0, 5, 10, 15, 30, and 60 min). The phosphorylation of EGFR was determined by Western blot with GAPDH as a loading control. Data are expressed as mean ± S.E.M. of three independent experiments (*n* = 3). ^#^ *p* < 0.05, as compared with the cells exposed to vehicle alone; or significantly different as indicated.

TNF-α-induced expression of various genes has been shown to be involved in the transactivation of EGFR [15,16]. To assess whether EGFR participated in TNF-α-mediated CCL20 secretion and gene expression, as shown in Figure 3A, TNF-α-induced CCL20 production was markedly reduced by AG1478 (an inhibitor of EGFR). Further, TNF-α-induced CCL20 mRNA expression and promoter activity were also inhibited by AG1478, determined by real-time PCR and the promoter–reporter assay, respectively (Figure 3B). To ensure the role of EGFR in CCL-20 expression, as shown in Figure 3C, transfection with EGFR siRNA downregulated EGFR protein expression and reduced TNF-α-induced CCL20 mRNA expression. We attempted to explore whether TNF-α-stimulated phosphorylation of EGFR was involved in TNF-α-mediated responses. Data in Figure 3D show that TNF-α time-dependently stimulated EGFR phosphorylation which was reduced by either TNFR nAb or AG1478 (Figure 3D). These results suggest that TNF-α-induced CCL20 expression is dependent on the activation of the TNFR1/EGFR cascade in HCFs.

### 2.4. Involvement of p38 MAPK in TNF-α-Induced CCL20 Expression

**Figure 4:**
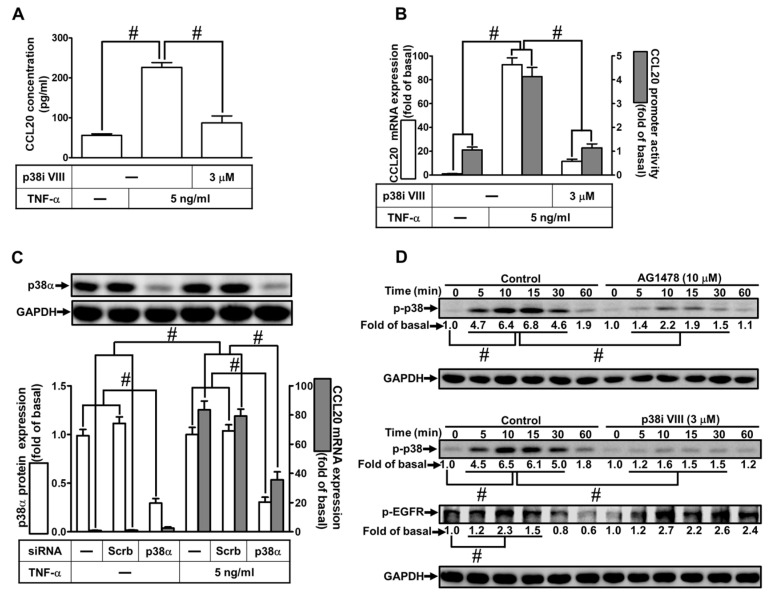
Involvement of p38 MAPK in TNF-α-induced CCL20 expression. (**A**) Cells were pretreated with 3 μM p38 inhibitor VIII (p38i VIII) for 1 h and then incubated with 5 ng/mL TNF-α for 12 h. The conditioned media were utilized to determine the CCL20 level via an ELISA kit. (**B**) Cells were pretreated with 3 μM p38i VIII for 1 h and then incubated with 5 ng/mL TNF-α for the indicated time. The mRNA levels and promoter activity of CCL20 were determined by real-time PCR (2 h) and promoter assay (4 h), respectively. (**C**) Cells were transfected with scrambled or p38α siRNA and then incubated with TNF-α for 2 h. The mRNA levels of CCL20 were determined by real-time PCR. The protein levels of p38α were determined by Western blot with GAPDH as a loading control. (**D**) Cells were pretreated without or with 10 μM AG1478 or 3 μM p38i VIII for 1 h and then treated with TNF-α for the indicated times (0, 5, 10, 15, 30, and 60 min). The phosphorylation of p38 and EGFR was determined by Western blot with GAPDH as a loading control. Data are expressed as mean ± S.E.M. of three independent experiments (*n* = 3). ^#^ *p* < 0.05, as compared with the cells exposed to vehicle alone; or significantly different as indicated.

TNF-α can stimulate p38 MAPK phosphorylation leading to the expression of various genes in different types of cells [4,17,24]. To investigate whether p38 MAPK was involved in TNF-α-induced CCL20 protein production and transcription activity, as shown in Figure 4A, pretreatment with an inhibitor of p38 MAPK inhibitor (p38i) VIII had a significant decrease in TNF-α-induced CCL20 secretion. In addition, the mRNA expression and transcriptional activity of CCL20 induced by TNF-α were inhibited by p38i VIII, determined by real-time PCR and luciferase report assay, respectively (Figure 4B). To ascertain the role of p38 MAPK in TNF-α-induced CCL20 expression, as shown in Figure 4C, transfection with p38α siRNA downregulated the p38α protein expression and reduced TNF-α-induced CCL20 mRNA expression. To differentiate the relationship between EGFR and p38 MAPK phosphorylation, as shown in Figure 4D, pretreatment with AG1478 or p38i VIII reduced TNF-α-stimulated p38 MAPK phosphorylation in HCFs, whereas p38i VIII had no significant effect on TNF-α-stimulated EGFR phosphorylation, implying that p38 MAPK was a downstream component of EGFR in TNF-α-mediated responses. These results suggest that TNF-α-induced CCL20 expression is mediated through TNFR1-dependent activation of the EGFR/p38 MAPK pathway in HCFs.

### 2.5. Involvement of JNK1/2 in TNF-α-Induced CCL20 Expression

**Figure 5:**
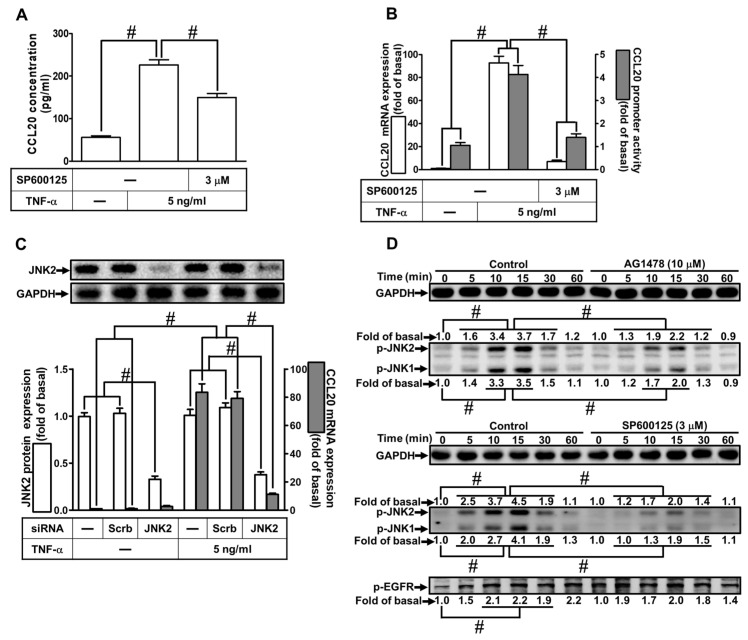
Involvement of JNK1/2 in TNF-α-induced CCL20 expression. (**A**) Cells were pretreated with 3 μM SP600125 for 1 h and then incubated with 5 ng/mL TNF-α for 12 h. The conditioned media were utilized to determine the CCL20 level via an ELISA kit. (**B**) Cells were pretreated with 3 μM SP60012 for 1 h and then incubated with 5 ng/mL TNF-α for the indicated time. The mRNA levels and promoter activity of CCL20 were determined by real-time PCR (2 h) and promoter assay (4 h), respectively. (**C**) Cells were transfected with scrambled or JNK2 siRNA and then incubated with TNF-α for 2 h. The mRNA levels of CCL20 were determined by real-time PCR. The protein levels of JNK2 were determined by Western blot with GAPDH as a loading control. (**D**) Cells were pretreated without or with 10 μM AG1478 or 3 μM SP600125 for 1 h and then treated with TNF-α for the indicated times (0, 5, 10, 15, 30, and 60 min). The phosphorylation of JNK1/2 and EGFR was determined by Western blot with GAPDH as a loading control. Data are expressed as mean ± S.E.M. of three independent experiments (*n* = 3). ^#^ *p* < 0.05, as compared with the cells exposed to vehicle alone; or significantly different as indicated.

Activation of JNK1/2 has been shown to trigger the expression of inflammatory proteins induced by TNF-α in various types of cells [4,17]. To determine whether JNK1/2 was involved in TNF-α-induced CCL20 protein secretion and transcription activity, as shown in Figure 5A, pretreatment with SP600125 (an inhibitor of JNK1/2) attenuated the TNF-α-induced CCL20 protein secretion. Moreover, TNF-α-induced CCL20 mRNA expression and promoter activity were attenuated by pretreatment with SP600125 (Figure 5B). To ensure the role of JNK1/2 in TNF-α-mediated responses, as shown in Figure 5C, transfection of HCFs with JNK2 siRNA knocked down JNK2 protein and attenuated TNF-α-induced CCL20 mRNA. To dissect the relationship between EGFR and JNK1/2 phosphorylation, as shown in Figure 5D, pretreatment with AG1478 or SP600125 reduced TNF-α-stimulated JNK1/2 phosphorylation in HCFs, whereas SP600125 had no significant effect on TNF-α-stimulated EGFR phosphorylation, implying that JNK1/2 was a downstream component of EGFR in TNF-α-mediated responses. These results suggest that TNF-α-induced CCL20 gene expression and secretion are mediated through the activation of the EGFR/JNK1/2 cascade in HCFs.

### 2.6. Involvement of FoxO1 in TNF-α-Induced CCL20 Expression

**Figure 6:**
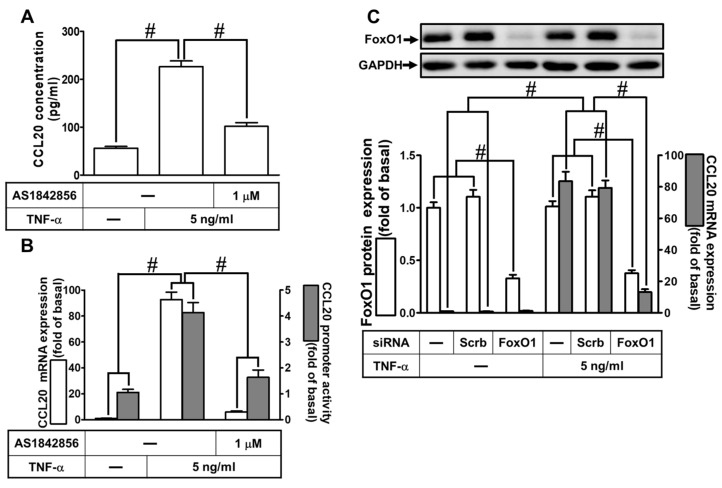

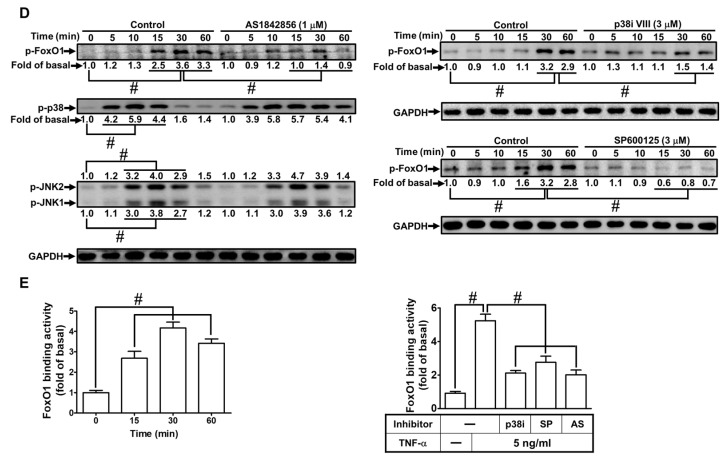
Involvement of FoxO1 in TNF-α-induced CCL20 expression. (**A**) Cells were pretreated with 1 μM AS1842856 for 1 h and then incubated with 5 ng/mL TNF-α for 12 h. The conditioned media were utilized to determine the CCL20 level via an ELISA kit. (**B**) Cells were pretreated with 1 μM AS1842856 for 1 h and then incubated with 5 ng/mL TNF-α for the indicated time. The mRNA levels and promoter activity of CCL20 were determined by real-time PCR (2 h) and promoter assay (4 h), respectively. (**C**) Cells were transfected with scrambled or FoxO1 siRNA and then incubated with TNF-α for 2 h. The mRNA levels of CCL20 were determined by real-time PCR. The protein levels of FoxO1 were determined by Western blot with GAPDH as a loading control. (**D**) Cells were pretreated without or with 1 μM AS1842856, 3 μM p38i VIII, or 3 μM SP600125 for 1 h and then treated with TNF-α for the indicated times (0, 5, 10, 15, 30, and 60 min). The phosphorylation of FoxO1, p38, or JNK1/2 was determined by Western blot with GAPDH as a loading control. (**E**) Cells were pretreated without or with 3 μM p38i VIII, 3 μM SP600125, or 1 μM AS1842856 for 1 h and then stimulated with TNF-α for the indicated time intervals or 30 min. The binding of FoxO1 to the promoter region of CCL20 was determined with a ChIP assay. Data are expressed as mean ± S.E.M. of three independent experiments (*n* = 3). ^#^ *p* < 0.05, as compared with the cells exposed to vehicle alone; or significantly different as indicated.

TNF-α-induced expression of inflammatory proteins has been reported to be mediated through the activation of intracellular signaling components and transcription factors such as FoxO1. To investigate whether FoxO1 regulated TNF-α-induced CCL20 protein production and gene expression, as shown in Figure 6A, pretreatment with AS1842856 (an inhibitor of FoxO1) decreased the TNF-α-induced CCL20 protein secretion. Further, TNF-α-induced CCL20 mRNA expression and promoter activity were also inhibited by AS1842856, determined by real-time PCR and report gene assay, respectively (Figure 6B). To reveal the role of FoxO1 in TNF-α-mediated responses, as shown in Figure 6C, transfection of HCFs with FoxO1 siRNA knocked down FoxO1 protein and attenuated TNF-α-induced CCL20 mRNA. To investigate the relationship among p38 MAPK, JNK1/2, and FoxO1 phosphorylation, as shown in Figure 6D, pretreatment with AS1842856, p38i VIII, or SP600125 reduced TNF-α-stimulated FoxO1 phosphorylation in HCFs. In addition, AS1842856 had no significant effect on TNF-α-stimulated p38 MAPK and JNK1/2 phosphorylation, implying that FoxO1 was a downstream component of p38 MAPK and JNK1/2. Data in Figure 6E show that TNF-α-stimulated FoxO1 phosphorylation time-dependently enhanced its association with the FoxO1 binding site on the CCL20 promoter, which was diminished by p38i VIII, SP600125, or AS1842856. These results reveal that TNF-α-induced CCL20 expression is mediated through the activation of p38 MAPK or JNK1/2/FoxO1 in HCFs.

### 2.7. Involvement of NF-κB p65 in TNF-α-Induced CCL20 Expression

**Figure 7:**
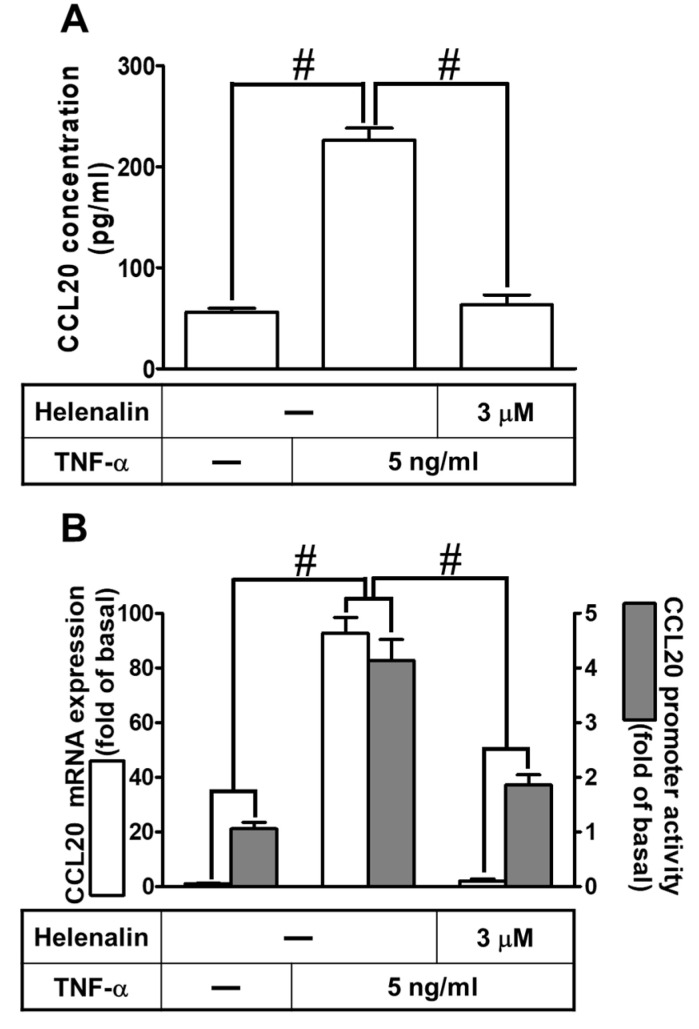

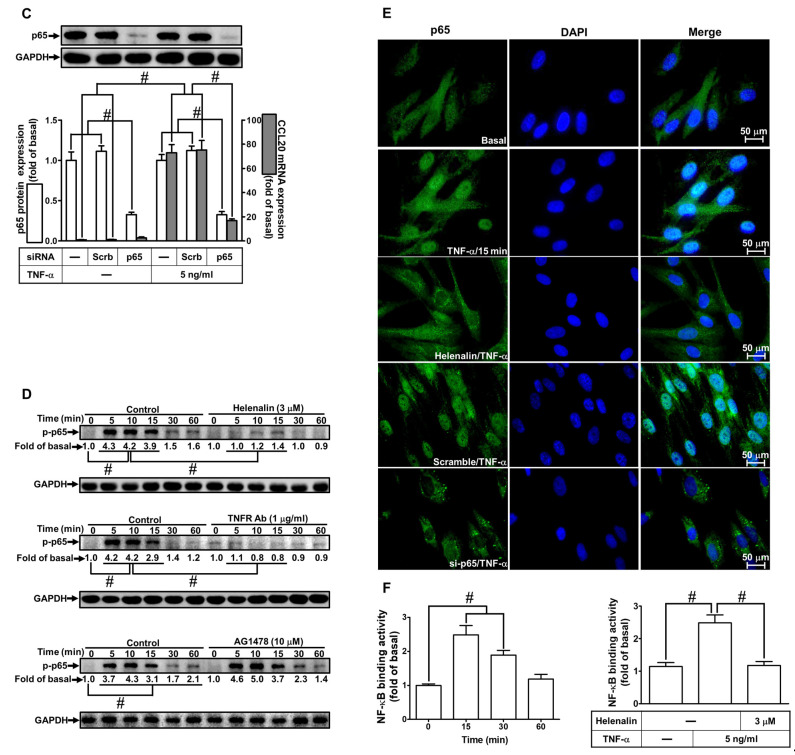
Involvement of NF-κB p65 in TNF-α-induced CCL20 expression. (**A**) Cells were pretreated with 3 μM helenalin for 1 h and then incubated with 5 ng/mL TNF-α for 12 h. The conditioned media were utilized to determine the CCL20 level via an ELISA kit. (**B**) Cells were pretreated with 3 μM helenalin for 1 h and then incubated with 5 ng/mL TNF-α for indicated time intervals. The mRNA levels and promoter activity of CCL20 were determined by real-time PCR (2 h) and promoter assay (4 h), respectively. (**C**) Cells were transfected with scrambled or p65 siRNA and then incubated with TNF-α for 2 h. The mRNA levels of CCL20 were determined by real-time PCR. The protein levels of p65 were determined by Western blot with GAPDH as a loading control. (**D**) Cells were pretreated without or with 3 μM helenalin, 1 μg/mL TNFR1 Ab, or 10 μM AG1478 for 1 h and then treated with TNF-α for the indicated times (0, 5, 10, 15, 30, and 60 min). The phosphorylation of p65 was determined by Western blot with GAPDH as a loading control. (**E**) Cells were pretreated without or with helenalin (3 μM) for 1 h or transfected with scrambled or p65 siRNA, and then incubated with TNF-α for 15 min. Cells were fixed and then labeled with an anti-phospho-p65 antibody and then a FITC-conjugated secondary antibody. The localization and expression of phospho-p65 were determined by immunofluorescent staining (green), and nuclei were stained with DAPI (blue). Scale bar: 50 µm. (**F**) Cells were pretreated without or with 3 μM helenalin for 1 h and then stimulated with TNF-α for the indicated time intervals or 15 min. The binding of NF-κB p65 to the promoter region of CCL20 was determined with a ChIP assay. Data are expressed as mean ± S.E.M. of three independent experiments (*n* = 3). ^#^ *p* < 0.05, as compared with the cells exposed to vehicle alone; or significantly different as indicated.

NF-κB is a master transcription factor involved in the expression of inflammatory proteins induced by TNF-α in different types of cells [4,25]. To determine whether NF-κB p65 is involved in the TNF-α-induced CCL20 gene expression and secretion, as shown in Figure 7A, pretreatment with Helenalin (an inhibitor of NF-κB) markedly reduced CCL20 secretion. Further, TNF-α-induced CCL20 mRNA expression and promoter activation were also attenuated by Helenalin, determined by real-time PCR and the reporter gene assay, respectively (Figure 7B). To verify that NF-κB was involved in TNF-α-mediated responses, as shown in Figure 7C, transfection with p65 siRNA downregulated the p65 protein expression and reduced TNF-α-induced CCL20 mRNA expression. Moreover, to determine whether NF-κB p65 phosphorylation is required for the TNF-α-induced responses, as shown in Figure 7D, TNF-α time-dependently stimulated phosphorylation of NF-κB p65, which was attenuated by pretreatment with Helenalin or TNFR1 nAb but not AG1478, which implied that EGFR did not participate in regulating the NF-κB activation. Activated NF-κB p65 was translocated into the nucleus, which was observed by immunofluorescent staining. We found that TNF-α stimulated translocation and phosphorylation of NF-κB p65 from the cytosol into the nucleus, which was attenuated by transfection with Helenalin or p65 siRNA (Figure 7E). To further ascertain whether p65 participated in TNF-α-regulated CCL20 transcriptional activity in HCFs, a ChIP assay was performed. As shown in Figure 7F, TNF-α increased NF-κB p65 activation and binding to the CCL20 promoter region, which was inhibited by pretreatment with Helenalin. These data suggest that TNF-α-induced CCL20 expression is mediated through NF-κB p65 phosphorylation/translocation and binding to the CCL20 promoter in HCFs.

## 3. Discussion

**Figure 8:**
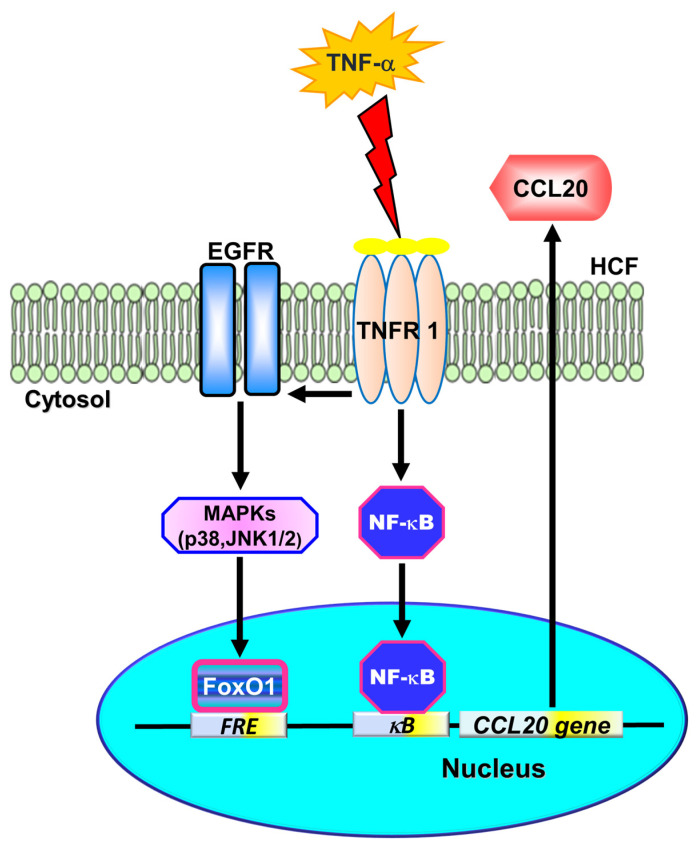
Schematic diagram illustrating the proposed signaling pathway involved in TNF-α-induced CCL20 expression and secretion in HCFs. TNF-α-induced CCL20 expression was, at least partially, mediated through binding to TNFR1 leading to transactivation of EGFR. Activated EGFR promoted the phosphorylation of p38 MAPK- or JNK1/2-dependent FoxO1 activation, which further bound with the FoxO1 response element (FRE) on the CCL20 promoter. In addition, TNF-α also turned on NF-κB transcription factors. Either FoxO1 or NF-κB activation could enhance the expression of CCL20 induced by TNF-α, which may be engaged in the inflammatory responses in HCFs. A better understanding of mechanisms underlying the regulation of the CCL20 gene by TNF-α will support more opportunities to develop anti-inflammatory therapeutic strategies for treating cardiac inflammation.

In ischemic heart disease, CCL20 has been demonstrated to have higher circulating levels [9]. Therefore, CCL20 could be a biomarker to predict HF severity and prognosis [10]. The severity of heart disease is also determined via the relationship between the circulating levels of TNF-α and functional HF classification [1,2,26]. Thus, CCL20-induction by stimulation with TNF-α may be a good marker associated with heart diseases. However, the possible mechanisms underlying TNF-α-induced CCL20 expression are still unknown. In this study, we demonstrated that TNF-α could induce CCL20 expression and secretion in HCFs. Moreover, our findings are the first to uncover that TNF-α-induced CCL20 expression is, at least partially, mediated by binding to TNFR1, leading to the transactivation of EGFR. Activated EGFR promoted the phosphorylation of p38 MAPK- or JNK1/2-dependent FoxO1 activation, which further bound the FoxO1 response element (FRE) to the CCL20 promoter. In addition, TNF-α also activated NF-κB transcription factors. Either FoxO1 or NF-κB activation could enhance the expression of CCL20 induced by TNF-α, which may be engaged in the inflammatory responses in HCFs. (Figure 8).

TNF-α is a pro-inflammatory cytokine that activates innate immune pathways and triggers inflammatory responses. It regulates several critical cellular functions, including cell proliferation, survival, apoptosis, and differentiation. TNF antagonism is cardioprotective in the in vivo model of HF [6,27]. Indeed, TNF-α acts through two transmembrane receptor subtypes: TNFR1 and TNFR2. TNFR1- or TNFR2-induced opposite remodeling responses were accompanied by the deprivation or mitigation of cardiac inflammation as assessed by NF-κB activation, inflammatory cytokine expression, MAPK activity, and apoptosis [3,4]. In most mammalian tissues, TNFR1 is constitutively expressed. Our results in HCFs are consistent with previous findings revealing that NF-κB and MAPKs are activated and mediated through TNFR1. In our data, TNF-α activates TNFR1 to stimulate downstream signaling components in HCFs.

RTKs play an important role in the control of most fundamental cellular processes, including the cell cycle, cell migration, cell metabolism, and survival, as well as cell proliferation and differentiation. During cell injury, the inhibition of EGFR also prevents the activation of inflammation, fibrosis, apoptosis, and oxidative stress pathways [28]. TNF-α increased EGFR expression and subsequent EGFR binding with EGF and heparin-binding EGF to enhance cyclooxygenase-2 (COX-2) expression in primary myofibroblasts isolated from human colon tissue [15]. Our previous report revealed that in HCFs, TNF-α induces vascular cell adhesion protein 1 expression via the c-Src-dependent transactivation of EGFR [16]. In HCFs, we also found that pretreatment with EGFR inhibitor (AG1478) can inhibit TNF-α-induced CCL20 expression. The engagement of EGFR in CCL20 expression may result from EGFR phosphorylation inhibited by TNFR-1 Ab or AG1478 pretreatment. These data suggest that TNF-α-induced CCL20 expression is mediated through the activation of the TNFR1/EGFR pathway in HCFs.

EGFR is a part of the signaling networks activated by various stimuli which are independent of EGF. Therefore, EGFR is involved in the cellular responses and signaling events initiated by these various stimuli. A diverse group of G protein-coupled receptors, adhesion receptors, and cytokine receptors can induce EGF-independent tyrosine phosphorylation of the EGFR [29]. It has been shown that the binding of TNF-α onto TNFR1 is considered to be an irreversible mechanism, whereas the binding of TNF-α onto TNFR2 has both rapid on and off kinetics [4]. Hobbs et al. demonstrated that in young adult mouse colon epithelial cells, TNF-α-induced protein and mRNA expression of COX-2 are inhibited by blockade of EGFR kinase activity or TNFR1 knockout [24]. Additionally, this TNF-α-induced COX-2 upregulation is also mediated through downstream p38 MAPK activity. Our results are consistent with these findings, indicating that TNF-α transactivates EGFR and downstream MAPKs signaling to upregulate inflammatory mediators in HCFs. All RTKs contain an extracellular ligand-binding domain that is usually glycosylated. The ligand-binding domain is connected to the cytoplasmic domain by a single transmembrane helix. The cytoplasmic domain contains a conserved protein tyrosine kinase core and additional regulatory sequences that are subjected to auto-phosphorylation and phosphorylation by heterologous protein kinases. EGFR transactivation could be activated by metalloproteinases-dependent heparin-binding EGF-like growth factor [30] or non-receptor tyrosine kinases activity [31,32]. However, whether TNF-α-induced CCL20 expression is mediated through the activation of NRTKs or transactivation of RTKs through a metalloproteinases-dependent mechanism is still unknown. It is worth evaluating in the future in HCFs.

Mitogen-activated protein kinases (MAPKs) regulate several kinds of cellular programs by relaying extracellular stimuli to intracellular responses. All eukaryotic cells have many MAPK pathways, which coordinately regulate gene expression, protein synthesis, metabolism, mitosis, motility, cell cycle machinery, survival, apoptosis, and differentiation. In mammals, MAPKs are characterized into seven groups. Conventional MAPKs include the extracellular signal-regulated kinases 1/2 (ERK1/2), JNK1/2/3, p38 isoforms (α, β, γ, and δ), and ERK5 [33]. So far, the most widely studied groups of mammalian MAPKs are the ERK1/2, JNKs, and p38 isoforms, but recent studies have shed light on the regulation and function of other groups of MAPKs [34]. Intracellular MAPK signaling cascades may play a key role in the pathogenesis of cardiac and vascular diseases [35]. Sopontammarak et al. indicated that in the volume overload model of hypertrophy, the activity of p38 MAPK was markedly increased by10-fold; however, in the chronic pressure overload model of hypertrophy, the activity of JNK1/2 was significantly increased [36]. Takeishi et al. also found that ERK1/2, p38 MAPK, big MAP kinase 1, and c-Src, are activated by acute mechanical stretch or chronic pressure-overload [37]. Our previous study indicated that TNF-α bound to TNFR1 and then activated PKCα, NADPH oxidase, and reactive oxygen species generation leading to activation of MAPKs [17]. Another study indicated that *Chlamydophila pneumoniae* infection induces the release of CCL20 through activation of p38 MAPK but not ERK1/2 in human bronchial epithelial cells [38]. It is controversial that various members of MAPKs may participate in the cellular functions regulated by different stimuli. Therefore, the involvement of MAPKs in the upregulation of CCL20 induced by TNF-α was investigated in HCFs. In the current study, we found that the inhibitors of p38 MAPK and JNK1/2, p38 inhibitor VIII and SP600125, individually inhibited CCL20 expression induced by TNF-α. On the other hand, AG1478 reduced the levels of phosphorylation of p38 MAPK and JNK1/2. In contrast, p38 inhibitor VIII and SP600125 could attenuate the phosphorylation of p38 MAPK and JNK1/2, respectively, but had no effect on EGFR phosphorylation stimulated by TNF-α. These data suggest that TNF-α-induced CCL20 expression is, at least in part, mediated through EGFR-dependent p38 MAPK or JNK1/2 pathway in HCFs.

The transcription factor FoxO1 has been reported to be activated by TNF-α in both in vitro and in vivo studies [39]. It was demonstrated that in high glucose, TNF-α-enhanced FoxO1 nuclear translocation leads to binding to the CCL20 promoter and turns on transcription [40]. In obese adipose, increased production of TNF-α results in a low-grade inflammation of adipose tissue from robust induction of pro-inflammatory cytokines mediated through FoxO1-dependent expression of CCAAT/enhancer-binding protein β, which in turn binds to the cytokine promoter [41]. Wang et al. also indicated that TNF-α activated the pro-apoptotic transcription factor FoxO1 in primary human adult dermal fibroblasts [42]. Moreover, Miao et al. revealed that overexpression of active FoxO1 strikingly upregulates CCL20 expression and secretion by TNF-α in HepG2 hepatoma cells [14]. Therefore, we investigated the role of FoxO1 in CCL20 expression induced by TNF-α in HCFs. In our study, TNF-α-induced CCL20 expression was mediated by FoxO1 phosphorylation, leading to its binding to the CCL20 promoter. TNF-α-stimulated FoxO1 phosphorylation and its binding activity were attenuated by the inhibitor of EGFR, JNK1/2, or p38 MAPK. These results indicate that in HCFs, TNF-α-induced CCL20 expression is mediated through the EGFR/p38 MAPK or JNK1/2/FoxO1 pathway.

NF-κB represents a family of inducible transcription factors which upregulate a series of genes, including cytokines and chemokines, which are involved in various processes of the inflammatory and immune responses. NF-κB has been implicated in the pathogenesis of many kinds of inflammatory disorders. They have also been linked to several pathologic processes in the myocardium, including hypertrophy, apoptosis, cardiomyocyte pro-inflammatory cytokine release, and ischemia/reperfusion injury [43]. CCL20 also contributes to the progression of many cancers by activating various signaling proteins, especially NF-κB [44]. In both diseased conditions and healthy organisms, TNF-α, a key mediator and regulator of mammalian immune responses, induces NF-κB and MAPK activities, constituting its major biochemical functions. A previous study suggested that in coronary artery disease individuals, the use of cholesterol-lowering drugs can significantly upregulate thrombomodulin gene expression, whereas TNF-α and NF-κB were significantly downregulated [45]. Targeting the TNF-α/NF-κB axis can be of clinical significance in the strategy of thromboembolic and inflammatory disorders. Therefore, we evaluated the role of NF-κB in CCL20 expression induced by TNF-α in HCFs. Our data show that TNF-α-induced CCL20 expression is mediated by NF-κB, and we also found that the inhibitor of NF-κB helenalin or TNFR1 nAb pretreatment inhibited NF-κB p65 phosphorylation. These results indicate that TNF-α-induced CCL20 expression is mediated by TNFR1/NF-κB p65 pathway in HCFs.

## 4. Materials and Methods

### 4.1. Materials

Dulbecco’s modified Eagle’s medium (DMEM)/F-12 and fetal bovine serum were purchased from Thermo Fisher Scientific (Grand Island, NY, USA). BioTrace^TM^ NT membrane was purchased from Pall Life Sciences (Ann Arbor, MI, USA). The enhanced chemiluminescence (ECL) Western blotting detection system was purchased from Perkin Elmer (Waltham, MA, USA). Anti-phospho-EGFR (Tyr^1173^, Cat#4407), anti-phospho-p38 MAPK (Thr^180^/Tyr^182^, Cat#9211), anti-phospho-JNK1/2 (Thr^183^/Tyr^185^, Cat#9255), anti-phospho-FoxO1 (Ser^256^, Cat#9461), and anti-phospho-p65 (Ser^536^, Cat#3033) antibodies were purchased from Cell Signaling (Danvers, MA, USA). Antibodies of anti-TNFR1 (Cat#sc-52739), anti-TNFR2 (Cat#sc-8041), and p38 MAPK inhibitor (p38i) VIII were purchased from Santa Cruz Biotechnology (Santa Cruz, CA, USA). Anti-GAPDH (Cat#MCA-1D4) was purchased from EnCor Biotechnology (Gainesville, FL, USA). AG1478, SP600125, and Tanshinone IIA were purchased from Enzo Life Science (Farmingdale, NY, USA). AS1842856 was obtained from EMD Millipore (Billerica, MA, USA). Helenalin was purchased from Cayman Chemical (Ann Arbor, MI, USA). Recombinant human TNF-α protein was purchased from R&D Systems (Minneapolis, MN, USA). TRIzol reagent, enzymes, and other chemicals were purchased from Sigma-Aldrich (St. Louis, MO, USA).

### 4.2. Human Cardiac Fibroblasts Cells Culture

HCFs isolated from the human heart were purchased from ScienCell Research Lab (San Diego, CA, USA). They were cultured in a DMEM/F-12 medium supplemented with 10% FBS and antibiotics (100 U/mL penicillin G, 100 μg/mL streptomycin, and 250 ng/mL fungizone) at 37 °C in a humidified 5% CO_2_ atmosphere using the method of Yang et al. [46]. When the cultures reached confluence, cells were treated with 0.05% (*w*/*v*) trypsin/0.53 mM EDTA for 1 min at 37 °C. The cell suspension was diluted with DMEM/F-12 containing 10% (*v*/*v*) FBS to a concentration of 2 × 10^5^ cells/mL. The cell suspension was seeded in (1 mL/well) 12-well culture plates, (2 mL/well) 6-well culture plates, and (10 mL/dish) 10 cm culture dishes and made quiescent at confluence by incubation in serum-free DMEM/F-12 for 24 h. The following experiments were carried out using HCF passages from 5 to 7.

### 4.3. Preparation of Cell Extracts and Western Blotting Analysis

We used the method of Yang et al. [46] for the preparation of cell extracts and Western blotting analysis. Growth-arrested cells were incubated without or with different concentrations of TNF-α at 37 °C for the indicated time intervals. When pharmacological inhibitors were applied, they were added for 1 h before exposure to TNF-α. After incubation, the cells were rapidly washed with ice-cold PBS. The lysates were dissolved in the sample buffer. The denatured proteins (15 μL/each) were subjected to SDS-PAGE using a 10% running gel. Proteins were transferred to nitrocellulose membranes which were incubated successively at room temperature with 5% (*w*/*v*) BSA in TTBS for 1 h. Membranes were incubated overnight at 4 °C with a primary antibody used at a dilution of 1:1000 in TTBS, washed several times by TTBS, and then incubated with 1:2000 dilution of an anti-rabbit or anti-mouse secondary antibody for 1 h. Following incubation, the membranes were washed comprehensively with TTBS. An anti-GAPDH antibody was used as an internal control. Immunoreactive bands on membranes were detected using ECL reagents and captured by a UVP BioSpectrum 500 Imaging System (Upland, CA, USA). UN-SCAN-IT gel software 7.1 (Silk Scientific, Orem, UT, USA) was used to quantify image densitometry.

### 4.4. Total RNA Extraction and Real-Time PCR Analysis

Total RNA was extracted with 0.5 mL/dish Trizol (Sigma-Aldrich, St. Louis, MO, USA) from HCFs treated with TNF-α for various time intervals using the method of Yang et al. [46]. When inhibitors were used, they were added 1 h before the application of TNF-α. First-strand cDNA synthesis was performed with 2 μg of total RNA using random hexamers as primers in a final volume of 20 μL (5 μg/μL random hexamers, 1 mM dNTPs, 2 units/μL RNase inhibitor, and 1 unit/μL of superscript II reverse transcriptase (Invitrogen™/Thermo Fisher Scientific, Carlsbad, CA, USA). The reaction was carried out at 37 °C for 60 min. Real-time PCR was performed with the LabStar Probe qPCR master mix (Taigen Bioscience Corp. Taipei, Taiwan) gene expression assay system using the method of Yang et al. [46]. The primers and probe mixtures were used for CCL20 and GAPDH. PCR was performed using a StepOnePlus™ Real-Time PCR System (Applied Biosystems™/Thermo Fisher Scientific, Foster City, CA, USA). The relative amount of the target gene was calculated using 2^(Ct test gene-Ct GAPDH)^ (Ct = threshold cycle). The primer sequences were as follows:

Human CCL20: (NM_001130046.2)

forward sequence: 5′-TCTGTGTGCGCAAATCCAA-3′;

reverse sequence: 5′-CCATTCCAGAAAAGCCACAGT-3′;

probe sequence: 5′-TGTGCGTCTCCTCAGTAA-3′.

Human GAPDH: (NM_001357943.2)

forward sequence: 5′-GCCAGCCGAGCCACAT-3′;

reverse sequence: 5′-CTTTACCAGAGTTAAAAGCAGCCC-3′;

probe sequence: 5′-CCAAATCCGTTGACTCCGACCTTCA-3′.

### 4.5. Measurement of Luciferase Promoter Activity

For the construction of the CCL20-luc plasmid, the human CCL20 promoter, a region spanning −817 to +33 bp [47], was cloned into a pGL3-basic vector. The CCL20-luc reporter construct was transiently transfected and the control pCMV-Gal encoding for β-galactosidase was present to normalize for transfection efficiency using the method of Yang et al. [46]. The luciferase activity was analyzed by a luciferase assay system (Promega, Madison, WI, USA). Firefly luciferase activities were standardized for β-galactosidase activity.

### 4.6. Transient Transfection with siRNA

HCFs were plated at 2 × 10^5^ cells/mL onto 12-well plates or 10 cm dishes until reaching about 70% confluence. Cells were washed once with PBS and then added to a 1 mL/well or 5 mL/dish of Opti-MEM medium (Gibco, Grand Island, NY, USA) before transfection using the method of Yang et al. [46]. The TNFR1 siRNA (SASI_Hs01_00033456; NM_001346092.2), p38 siRNA (HSS102352, HSS102353, HSS175313; NM_139012.3), JNK2 siRNA (HSS108550; NM_001364610.2), FoxO1 (SASI_Hs01_0076732; NM_002015.4), and p65 siRNA (SASI_ Hs01_00171091; NM_001404662.1) were obtained from Sigma (St. Louis, MO, USA). EGFR siRNA (sense:5′-GAAGGAAACUGAAUUCAAA-3′; anti-sense:5′-UUUGAAUUCAGUUUCCUU C-3′; NM_001346941.2) was purchased from MDBio Inc. (Taipei, Taiwan). Transient transfection of siRNAs was carried out using Lipofectamine 2000 transfection reagent (Invitrogen, Carlsbad, CA, USA). The DNA Lipofectamine reagent complexes were added to each well to a final concentration of 100 nM siRNA and then incubated at 37 °C for 5 h. After transfection, cells were incubated with DMEM/F-12 medium containing 10% FBS for an additional 3 h and then washed twice with PBS and maintained in serum-free DMEM/F-12 for 16 h before treatment with TNF-α.

### 4.7. Measurement of CCL20 Secretion

Secreted CCL20 protein levels in culture media of HCFs stimulated with TNF-α were quantified using ELISA development kits (https://www.rndsystems.com/products/human-ccl20-mip-3-alpha-quantikine-elisa-kit_dm3a00 (accessed on 1 May 2020)) according to the instructions of the manufacturer (R&D Systems, Minneapolis, MN, USA). The levels of the optical densities were determined immediately using a microplate reader set to 450 nm (SynergyH1 Hybrid Reader, BioTek, Winooski, VT, USA).

### 4.8. Immunofluorescence Staining

HCFs were cultured in 6-well culture plates with coverslips until reaching about 70% confluence and were serum-free for 24 h. Growth-arrested cells were transfected without or with scrambled and p65 siRNA and then stimulated with TNF-α (5 ng/mL) for the indicated time intervals. The cells were fixed, permeabilized, and stained using anti-phospho-NF-κB p65 antibody (1:200 dilutions) and 4′,6-diamidino-2-phenylindole (DAPI) after washing with ice-cold PBS using the method of Yang et al. [48]. The staining was successively followed by washing three times with PBS, incubation for 1 h with fluorescein isothiocyanate (FITC)-conjugated goat anti-mouse antibody (1:100 dilution) with 1% BSA, and washing three times with PBS. The images of phospho-p65 and nucleus were detected with a fluorescence microscope (Axiovert 200 M, Zeiss, Jena, Germany).

### 4.9. Chromatin Immunoprecipitation (ChIP) Assay

To detect the association of nuclear proteins with human CCL20 promoter, HCFs in a 10 cm dish were grown to confluence and serum-starved for 24 h. After treatment with TNF-α (5 ng/mL), protein–DNA complexes were fixed by 1% formaldehyde in the medium using the method of Yang et al. [48]. The fixed cells were washed and lysed in an SDS-lysis buffer. The cell lysates were sonicated at 4 °C and rotated for 30 min at 4 °C. After precleared, the sample was centrifuged, and the supernatant was transferred to a new tube. The concentrations of samples were quantified and balanced. One portion of the samples was used as DNA input controls, and the remains were subdivided into several portions and then incubated with anti-NF-κB and anti-FoxO1 antibodies overnight at 4 °C. The immunoprecipitating complexes of antibody–protein–DNA were collected using agarose protein A beads overnight with rotation at 4 °C. After incubation, the samples were successively washed with a low-salt buffer, high-salt buffer, LiCi buffer, and then eluted with elution buffer. The cross-linking of protein–DNA complexes was reversed by incubation at 65 °C overnight. The DNA was extracted and resuspended in H_2_O and subjected to Real-time PCR amplification performed with the SYBR GREEN assay system (New England Biolabs, Ipswich, MA, USA) with the primers of CCL20 described below. PCR was performed using a StepOnePlus™ Real-Time PCR System (Applied Biosystems™/Thermo Fisher Scientific, Foster City, CA, USA). The relative amount of the target gene was calculated using 2^(Ct test gene-Ct GAPDH)^ (Ct = threshold cycle). The primer sequence is as follows:

Human CCL20 ChIP qPCR sequences (NC_000002.12):

Forward: 5′-CCTGTGTGGGGCTGACCTTTGTATCG-3′;

Reverse: 5′-GGAGCAAACTCTTGGTACAGCACATGG-3′.

### 4.10. Statistical Analysis of Data

GraphPad Prizm Program 6.0 software (GraphPad, San Diego, CA, USA) was used as a tool for statistical analysis. We used the methods of statistical analysis previously described by Yang et al. [46]. We used one-way ANOVA followed by Dunnett’s post hoc test when comparing more than two groups of data or nonparametric Kruskal–Wallis test, followed by Dunn’s multiple comparison test when comparing multiple independent groups and the ANOVA normality assumptions not met. Post hoc tests were run only if F achieved *p* < 0.01, and there was no significant variance inhomogeneity. *p* values < 0.01 were considered to be statistically significant. Error bars were omitted when they fell within the dimensions of the symbols. All the data were expressed as mean ± SEM in three individual experiments (*n* = 3).

## 5. Conclusions

In the present report, we concluded that TNF-α-induced CCL20 expression is, at least partially, mediated through binding to TNFR1, leading to transactivation of EGFR. Activated EGFR promoted the phosphorylation of p38 MAPK- or JNK1/2-dependent FoxO1 activation, which further bound with the FoxO1 response element (FRE) on the CCL20 promoter. In addition, TNF-α also activated NF-κB transcription factors. Either FoxO1 or NF-κB activation could enhance the expression of CCL20 induced by TNF-α, which may be engaged in the inflammatory responses in HCFs. A better understanding of mechanisms underlying the regulation of the CCL20 gene by TNF-α will support more opportunities to develop anti-inflammatory therapeutic strategies for treating cardiac inflammation. However, the limitation of the present study is the lack of in vivo data. It is worth exploring animal models further in the future.

## Data Availability

The data presented in this study are available on request from the corresponding author.

## References

[1] Feldman A.M., Combes A., Wagner D., Kadakomi T., Kubota T., Li Y.Y., McTiernan C. (2000). The role of tumor necrosis factor in the pathophysiology of heart failure. J. Am. Coll. Cardiol..

[2] Torre-Amione G., Kapadia S., Benedict C., Oral H., Young J.B., Mann D.L. (1996). Proinflammatory cytokine levels in patients with depressed left ventricular ejection fraction: A report from the Studies of Left Ventricular Dysfunction (SOLVD). J. Am. Coll. Cardiol..

[3] Hamid T., Gu Y., Ortines R.V., Bhattacharya C., Wang G., Xuan Y.T., Prabhu S.D. (2009). Divergent tumor necrosis factor receptor-related remodeling responses in heart failure: Role of nuclear factor-kappaB and inflammatory activation. Circulation.

[4] Parameswaran N., Patial S. (2010). Tumor necrosis factor-α signaling in macrophages. Crit. Rev. Eukaryot. Gene Expr..

[5] Torre-Amione G., Kapadia S., Lee J., Durand J.B., Bies R.D., Young J.B., Mann D.L. (1996). Tumor necrosis factor-alpha and tumor necrosis factor receptors in the failing human heart. Circulation.

[6] Jobe L.J., Meléndez G.C., Levick S.P., Du Y., Brower G.L., Janicki J.S. (2009). TNF-alpha inhibition attenuates adverse myocardial remodeling in a rat model of volume overload. Am. J. Physiol. Heart Circ. Physiol..

[7] Westermann D., Van Linthout S., Dhayat S., Dhayat N., Schmidt A., Noutsias M., Song X.Y., Spillmann F., Riad A., Schultheiss H.P. (2007). Tumor necrosis factor-alpha antagonism protects from myocardial inflammation and fibrosis in experimental diabetic cardiomyopathy. Basic Res. Cardiol..

[8] Schutyser E., Struyf S., Van Damme J. (2003). The CC chemokine CCL20 and its receptor CCR6. Cytokine Growth Factor Rev..

[9] Safa A., Rashidinejad H.R., Khalili M., Dabiri S., Nemati M., Mohammadi M.M., Jafarzadeh A. (2016). Higher circulating levels of chemokines CXCL10, CCL20, and CCL22 in patients with ischemic heart disease. Cytokine.

[10] Hage C., Michaëlsson E., Linde C., Donal E., Daubert J.C., Gan L.M., Lund L.H. (2017). Inflammatory biomarkers predict heart failure severity and prognosis in patients with heart failure with preserved ejection fraction: A holistic proteomic approach. Circ. Cardiovasc. Genet..

[11] Iwamoto S., Kido M., Aoki N., Nishiura H., Maruoka R., Ikeda A., Okazaki T., Chiba T., Watanabe N. (2013). TNF-α is essential in the induction of fatal autoimmune hepatitis in mice through upregulation of hepatic CCL20 expression. Clin. Immunol..

[12] Chen P., Zhong Z., Jiang H., Chen H., Lyu J., Zhou L. (2019). Th17-associated cytokines multiplex testing indicates the potential of macrophage inflammatory protein-3 alpha in the diagnosis of biliary atresia. Cytokine.

[13] Zhao L., Xia J., Wang X., Xu F. (2014). Transcriptional regulation of CCL20 expression. Microbes Infect..

[14] Miao H., Zhang Y., Lu Z., Yu L., Gan L. (2012). FOXO1 increases CCL20 to promote NF-κB-dependent lymphocyte chemotaxis. Mol. Endocrinol..

[15] Yoo J., Rodriguez Perez C.E., Nie W., Edwards R.A., Sinnett-Smith J., Rozengurt E. (2012). TNF-α induces upregulation of EGFR expression and signaling in human colonic myofibroblasts. Am. J. Physiol. Gastrointest. Liver Physiol..

[16] Lin C.C., Pan C.S., Wang C.Y., Liu S.W., Hsiao L.D., Yang C.M. (2015). Tumor necrosis factor-alpha induces VCAM-1-mediated inflammation via c-Src-dependent transactivation of EGF receptors in human cardiac fibroblasts. J. Biomed. Sci..

[17] Lin C.C., Yang C.C., Wang C.Y., Tseng H.C., Pan C.S., Hsiao L.D., Yang C.M. (2015). NADPH oxidase/ROS-dependent VCAM-1 induction on TNF-α-challenged human cardiac fibroblasts enhances monocyte adhesion. Front. Pharmacol..

[18] Krenning G., Zeisberg E.M., Kalluri R. (2010). The origin of fibroblasts and mechanism of cardiac fibrosis. J. Cell Physiol..

[19] Souders C.A., Bowers S.L., Baudino T.A. (2009). Cardiac fibroblast: The renaissance cell. Circ. Res..

[20] Jiang Z.S., Jeyaraman M., Wen G.B., Fandrich R.R., Dixon I.M., Cattini P.A., Kardami E. (2007). High- but not low-molecular weight FGF-2 causes cardiac hypertrophy in vivo; possible involvement of cardiotrophin-1. J. Mol. Cell Cardiol..

[21] Ma Z.G., Yuan Y.P., Wu H.M., Zhang X., Tang Q.Z. (2018). Cardiac fibrosis: New insights into the pathogenesis. Int. J. Biol. Sci..

[22] Lindner D., Zietsch C., Tank J., Sossalla S., Fluschnik N., Hinrichs S., Maier L., Poller W., Blankenberg S., Schultheiss H.P. (2014). Cardiac fibroblasts support cardiac inflammation in heart failure. Basic Res. Cardiol..

[23] Thottakara T., Dhople V.M., Voss S., Schoen J., Voelker U., Blankenberg S., Westermann D., Hammer E., Lindner D. (2020). The Cardiac Fibroblast in heart failure—An inflammatory cell. Eur. Heart J..

[24] Hobbs S.S., Goettel J.A., Liang D., Yan F., Edelblum K.L., Frey M.R., Mullane M.T., Polk D.B. (2011). TNF transactivation of EGFR stimulates cytoprotective COX-2 expression in gastrointestinal epithelial cells. Am. J. Physiol. Gastrointest. Liver Physiol..

[25] Yang C.M., Lee I.T., Hsu R.C., Chi P.L., Hsiao L.D. (2013). NADPH oxidase/ROS-dependent PYK2 activation is involved in TNF-α-induced matrix metalloproteinase-9 expression in rat heart-derived H9c2 cells. Toxicol. Appl. Pharmacol..

[26] Koller-Strametz J., Pacher R., Frey B., Kos T., Woloszczuk W., Stanek B. (1998). Circulating tumor necrosis factor-alpha levels in chronic heart failure: Relation to its soluble receptor II, interleukin-6, and neurohumoral variables. J. Heart Lung Transpl..

[27] Moe G.W., Marin-Garcia J., Konig A., Goldenthal M., Lu X., Feng Q. (2004). In vivo TNF-alpha inhibition ameliorates cardiac mitochondrial dysfunction, oxidative stress, and apoptosis in experimental heart failure. Am. J. Physiol. Heart Circ. Physiol..

[28] Skibba M., Qian Y., Bao Y., Lan J., Peng K., Zhao Y., Zhong P., Hu J., Li X., Liang G. (2016). New EGFR inhibitor, 453, prevents renal fibrosis in angiotensin II-stimulated mice. Eur. J. Pharmacol..

[29] Carpenter G. (1999). Employment of the epidermal growth factor receptor in growth factor-independent signaling pathways. J. Cell Biol..

[30] Kalmes A., Daum G., Clowes A.W. (2001). EGFR transactivation in the regulation of SMC function. Ann. N. Y. Acad. Sci..

[31] Parsons J.T., Parsons S.J. (1997). Src family protein tyrosine kinases: Cooperating with growth factor and adhesion signaling pathways. Curr. Opin. Cell Biol..

[32] Shah B.H., Catt K.J. (2002). Calcium-independent activation of extracellularly regulated kinases 1 and 2 by angiotensin II in hepatic C9 cells: Roles of protein kinase C δ, Src/proline-rich tyrosine kinase 2, and epidermal growth receptor trans-activation. Mol. Pharm..

[33] Kyriakis J.M., Avruch J. (2012). Mammalian MAPK signal transduction pathways activated by stress and inflammation: A 10-year update. Physiol. Rev..

[34] Cargnello M., Roux P.P. (2011). Activation and function of the MAPKs and their substrates, the MAPK-activated protein kinases. Microbiol. Mol. Biol. Rev..

[35] Muslin A.J. (2008). MAPK signaling in cardiovascular health and disease: Molecular mechanisms and therapeutic targets. Clin. Sci..

[36] Sopontammarak S., Aliharoob A., Ocampo C., Arcilla R.A., Gupta M.P., Gupta M. (2005). Mitogen-activated protein kinases (p38 and c-Jun NH2-terminal kinase) are differentially regulated during cardiac volume and pressure overload hypertrophy. Cell Biochem. Biophys..

[37] Takeishi Y., Huang Q., Abe J., Glassman M., Che W., Lee J.D., Kawakatsu H., Lawrence E.G., Hoit B.D., Berk B.C. (2001). Src and multiple MAP kinase activation in cardiac hypertrophy and congestive heart failure under chronic pressure-overload: Comparison with acute mechanical stretch. J. Mol. Cell Cardiol..

[38] Kim T.-B., Moon K., Lee K.-Y., Park C.-S., Bae Y.-J., Moon H.-B., Cho Y.S. (2009). Chlamydophila pneumoniae triggers release of CCL20 and vascular endothelial growth factor from human bronchial epithelial cells through enhanced intracellular oxidative stress and MAPK activation. J. Clin. Immunol..

[39] Alikhani M., Alikhani Z., Graves D.T. (2005). FOXO1 functions as a master switch that regulates gene expression necessary for tumor necrosis factor-induced fibroblast apoptosis. J. Biol. Chem..

[40] Zhang C., Ponugoti B., Tian C., Xu F., Tarapore R., Batres A., Alsadun S., Lim J., Dong G., Graves D.T. (2015). FOXO1 differentially regulates both normal and diabetic wound healing. J. Cell Biol..

[41] Ito Y., Daitoku H., Fukamizu A. (2009). Foxo1 increases pro-inflammatory gene expression by inducing C/EBPbeta in TNF-alpha-treated adipocytes. Biochem. Biophys. Res. Commun..

[42] Wang X.W., Yu Y., Gu L. (2014). Dehydroabietic acid reverses TNF-α-induced the activation of FOXO1 and suppression of TGF-β1/Smad signaling in human adult dermal fibroblasts. Int. J. Clin. Exp. Pathol..

[43] Hall G., Hasday J.D., Rogers T.B. (2006). Regulating the regulator: NF-kappaB signaling in heart. J. Mol. Cell Cardiol..

[44] Chen W., Qin Y., Liu S. (2020). CCL20 Signaling in the Tumor Microenvironment. Adv. Exp. Med. Biol..

[45] Rafiq M., Liaquat A., Saeed N., Shamshad G.U., Mumtaz S., Khan M.J. (2020). Gene expression of thrombomodulin, TNF-α, and NF-KB in coronary artery disease patients of Pakistan. Mol. Biol. Rep..

[46] Yang C.C., Hsiao L.D., Lin H.H., Tseng H.C., Situmorang J.H., Leu Y.L., Yang C.M. (2020). Induction of HO-1 by 5, 8-dihydroxy-4′,7-dimethoxyflavone via activation of ROS/p38 MAPK/Nrf2 attenuates thrombin-induced connective tissue growth factor expression in human cardiac fibroblasts. Oxid. Med. Cell Longev..

[47] Kwon J.H., Keates S., Simeonidis S., Grall F., Libermann T.A., Keates A.C. (2003). ESE-1, an enterocyte-specific Ets transcription factor, regulates MIP-3alpha gene expression in Caco-2 human colonic epithelial cells. J. Biol. Chem..

[48] Yang C.-C., Hsiao L.-D., Shih Y.-F., Hsu C.-K., Hu C.-Y., Yang C.-M. (2022). Thrombin Induces COX-2 and PGE_2_ Expression via PAR1/PKCalpha/MAPK-Dependent NF-kappaB Activation in Human Tracheal Smooth Muscle Cells. Mediat. Inflamm..

